# Eligibility for Lung Cancer Screening in Switzerland: A Comparative Analysis of Three Data Sources From Lausanne and the Canton of Vaud

**DOI:** 10.3389/ijph.2026.1609104

**Published:** 2026-01-21

**Authors:** Louis Gros, Cynthia Schneider, May-Lucie Meyer, Julie Korber, Yves Henchoz, Julien Vaucher, Pedro Marques-Vidal, Kevin Ten Haaf, Chiara Pozzessere, Cédric Bongard, Christophe Von Garnier, Jean-Luc Bulliard, Kevin Selby

**Affiliations:** 1 Department of Oncology, Centre Hospitalier Universitaire Vaudois (CHUV), Lausanne, Switzerland; 2 Center for Primary Care and Public Health (Unisanté), University of Lausanne, Lausanne, Switzerland; 3 Department of Medicine and Specialties, Internal Medicine, Fribourg Hospital and University of Fribourg, Fribourg, Switzerland; 4 Department of Medicine, Internal Medicine, Lausanne University Hospital (CHUV) and University of Lausanne, Lausanne, Switzerland; 5 Department of Public Health, Erasmus MC-University Medical Center Rotterdam, Rotterdam, Netherlands; 6 Department of Diagnostic and Interventional Radiology, Lausanne University Hospital (CHUV), Lausanne, Switzerland; 7 Division of Pulmonology, Department of Medicine, Lausanne University Hospital (CHUV), Lausanne, Switzerland

**Keywords:** health disparities, lung cancer, screening eligibility, smoking cessation, Switzerland

## Abstract

**Objectives:**

This study aimed to estimate the proportion of individuals potentially eligible for lung cancer screening in Lausanne, canton of Vaud, Switzerland, and its associations with key sociodemographic and health-related covariates.

**Methods:**

We analyzed self-reported, cross-sectional data from three sources: Lausanne cohort 65+ (2014; *n* = 1,678; ages 65–70), CoLaus|PsyCoLaus (2014–2017; *n* = 3,839; ages 50–79), and the Swiss Health Survey (2022, representative of Vaud, ages 50–79). Eligibility was defined by the 2021 United States Preventive Services Task Force criteria. Screening eligibility prevalence was estimated separately in each dataset, and eligible and non-eligible participants were compared using bivariate hypothesis tests.

**Results:**

Eligibility was 18.2% in the Lc65+ cohort, 16.0% in CoLaus, and 14.4% in the Swiss Health Survey. Among eligible individuals, the prevalence of current smoking was 58.7% in Lc65+, 60.1% in the Swiss Health Survey, and 64.9% in CoLaus. Eligible participants tended to have a higher burden of comorbidities and social vulnerabilities, including cardiovascular disease, metabolic syndrome, depression, and lower education or income; statistically significant differences varied by dataset.

**Conclusion:**

In this Swiss population, about one in six adults met lung cancer screening criteria. Findings highlight the need for combining early detection with tobacco cessation, health promotion, and equitable access to care.

## Introduction

Lung cancer is the leading cause of cancer-related death worldwide [1]. In Switzerland alone, it accounts for approximately 3,250 deaths each year [2, 3]. Tobacco smoking remains the most important modifiable risk factor [4]. Prevention and early detection are thus key strategies to reduce the burden of lung cancer [5].

The Early Lung Cancer Action Program (ELCAP), launched in 1992, demonstrated that annual low-dose computed tomography (LDCT) screening enables detection of over 80% of lung cancers at stage I, with a 20-year lung cancer–specific survival exceeding 80% [6, 7]. Subsequently, in 2002 the U.S. National Lung Screening Trial (NLST), a randomized trial comparing low-dose CT to chest radiography, confirmed that low-dose CT screening reduces lung cancer mortality [8]. The Dutch-Belgian Lung Cancer Screening Trial (NELSON), a population-based, randomized controlled study, demonstrated a significant 24% mortality reduction compared with no screening [9].

Based on this evidence, lung cancer screening programs have been implemented in several countries worldwide [10]. In 2022, the Swiss Cancer Screening Committee recommended lung cancer screening, suggesting the implementation of organized programs [11]. Following this input, the Canton Vaud Health Authorities supported a 4-year pilot program run by the Lausanne University Hospital (CHUV) and the University Center for Primary Care and Public Health (Unisanté) [12]. This initiative aimed to assess the feasibility and acceptability of a structured hospital-based lung cancer screening program in the canton [12]. This was particularly relevant given persistently low participation in the United States and only recent improvements in the United Kingdom [13, 14].

Organized cancer screening programs rely on a well-defined target population for planning and adherence to multiple quality assessment criteria [15]. Accurately identifying this population is essential to ensure effective and equitable implementation [16]. Unlike screening programs for colorectal, breast, or cervical cancer, lung cancer screening is currently recommended only for individuals at high risk. Traditionally, eligibility criteria for lung cancer screening consider factors such as age, cumulative tobacco exposure (pack-years) and time since cessation of smoking [15]. However, eligibility criteria can be further refined to better target individuals most likely to benefit from screening. Risk-prediction models (such as PLCOm2012 or LLP version 3) can identify more future lung cancers and deaths without screening a larger number of individuals, although they require more detailed individual-level data [17, 18]. Owing to their simplicity and practicality, most national and pilot programs continue to use categorical rather than model-based eligibility criteria.

Cancer and notably lung cancer places a heavier burden on socioeconomically disadvantaged populations [19, 20]. Understanding and addressing these inequities is crucial for the equitable implementation of organized screening programs.

We hypothesized that a significant share of Lausanne and Vaud residents would meet eligibility criteria and that eligible individuals would differ meaningfully from their ineligible counterparts—particularly in terms of greater socioeconomic vulnerability and comorbidity burden.

This study aimed to estimate the proportion of Lausanne and Vaud residents eligible for lung cancer screening under the 2021 USPSTF criteria and to compare the demographic and clinical characteristics of eligible and ineligible individuals.

## Methods

We conducted cross-sectional analyses using three independent sources. Eligibility was defined according to the 2021 USPSTF criteria: individuals aged 50–79 years with a smoking history of at least 20 pack-years, who are either current smokers or former smokers who quit within the past 15 years [15]. These criteria used for the Vaud pilot project and were therefore selected to ensure alignment between population-based estimates and the local screening implementation, while remaining applicable across all three datasets.

### Data Sources

The three data sources included: 1) the Lausanne Cohort 65+ (Lc65+), designed to advance epidemiological and public health research on ageing, with a focus on the determinants, manifestations, and outcomes of frailty; 2) the CoLaus|PsyCoLaus (CoLaus) study, initiated in 2003 to improve understanding of the epidemiology and genetic determinants of cardiovascular risk factors and diseases in the Swiss population; and 3) the 2022 Swiss Health Survey (SHS), which provided representative data on health behaviors, lifestyle, and healthcare use among residents aged 15 years and older in Switzerland, using data from the canton of Vaud (of which Lausanne is the capital). These cohorts were selected for their data availability and complementarity, enabling a comprehensive assessment of screening eligibility [21, 22].

### Lausanne Cohort 65+ (Lc65+)

The Lc65+ study is an ongoing longitudinal cohort of community-dwelling adults aged 65 years and older living in Lausanne [21].

The Lc65+ study comprises three samples recruited at 5-year intervals (2004, 2009, and 2014) through simple random sampling from the population register of the canton of Vaud, Switzerland. Eligible participants were community-dwelling residents of Lausanne aged 65–69 years at the beginning of each recruitment year, while institutionalized individuals and those unable to respond due to cognitive impairment were excluded. Invitations were mailed with study information and a questionnaire, followed by two reminders, and participants were subsequently invited to in-person assessments at the study center. For the present analysis, we used data from the third recruitment wave (2014), which included 1,678 participants (participation rate 45.9%) after exclusion of 141 ineligible individuals who met the study exclusion criteria, namely, institutionalization or inability to respond due to cognitive impairment.

Variables available for subsequent comparisons between eligible and non-eligible participants included sex, age, country of birth, monthly income, indicators of financial vulnerability (financial assistance, financial difficulties, health insurance subsidy, or additional welfare), smoking status (current, former or never), alcohol problems, and major comorbidities (overweight, hypertension, hypercholesterolemia, coronary heart disease, stroke, diabetes, previous cancer, depression, arthrosis, or none of the above). Self-reported fatigue over the previous 4 weeks (no at all, a little, a lot) and educational level (from basic compulsory schooling to university or college degree) were also assessed.

The Lc65+ study protocol (no. 19/04), along with periodic updates, received approval from the Ethics Committee for Human Research of the Canton of Vaud. All participants provided written informed consent.

### CoLaus|PsyCoLaus

A detailed description of the recruitment procedure and follow-up process for the CoLaus study has been previously published [22]. Briefly, between 2003 and 2006, a representative, non-stratified sample of residents aged 35–75 years was recruited. The complete list of city residents (n = 56,694 in 2003) was obtained from the municipal population register, which includes all individuals residing in Lausanne for more than 90 days. A simple, non-stratified random sample of 19,830 residents (35% of the source population) was drawn, and invitation letters were mailed between June 2003 and May 2006. After two reminder letters and follow-up phone calls, 8,121 individuals agreed to participate, corresponding to a participation rate of 41% among those sampled and 57% among eligible responders. Of these, 6,738 attended the clinic and completed the baseline assessment; 549 (8.1%) non-Caucasian participants were excluded, resulting in a final baseline sample of 6,189 participants meeting all inclusion criteria.

The baseline examination was conducted between June 2003 and May 2006, followed by follow-up waves held from April 2009 to September 2012 (n = 5,064) and from May 2014 to April 2017 (n = 4,881). The median follow-up duration was 5.4 years (mean 5.6, range 4.5–8.8) for the first wave and 10.7 years (mean 10.9, range 8.8–13.6) for the second.

As the initial consent did not cover this type of analysis, we used data from the second follow-up, conducted between May 2014 and April 2017.

Variables available for subsequent comparisons between eligible and non-eligible participants included sex, age, and country of birth, as well as smoking status and alcohol consumption, along with clinical parameters and self-reported health measures. Clinical parameters comprised body mass index (BMI), hypertension, hypercholesterolemia, metabolic syndrome, diabetes, and sleep apnea. Additional health indicators included abdominal obesity (WHO definition), HDL and LDL cholesterol, triglycerides, and systolic and diastolic blood pressure. Self-reported measures encompassed overall health rating (very good, good, bad, or very bad), perceived fatigue during the previous 4 weeks (not at all, a little, or a lot), self-care difficulty (no difficulty, some difficulty, or requiring help), and weight loss over the previous 12 months.

### Swiss Health Survey

The SHS are nationwide cross-sectional surveys conducted every 5 years since 1992 by the Federal Statistical Office. They collect nationally representative data on residents aged 15 years and older, including information on health status, lifestyle behaviors, substance use, physical activity, health insurance, and healthcare utilization. Data are gathered via computer-assisted telephone interviews conducted by trained personnel.

The SHS did not collect exact quit dates but asked participants to indicate the time since smoking cessation using predefined categories, with the longest being “10 years or more.” Additionally, pack-year history is unavailable for former smokers in the SHS dataset.

For this study, we used tobacco-related data from the 2022 SHS, restricted to respondents residing in the canton of Vaud. The use of anonymized SHS data is granted upon request by the Federal Statistical Office and does not require ethics committee approval.

Variables available for analyses comparing eligible and non-eligible participants included sociodemographic, lifestyle, and health-related characteristics. These comprised age, sex, nationality, civil status, household income, household size, education, employment status and health indicators. Health indicators comprised self-rated health, long-term illness, and self-reported comorbidities (hypertension, high blood glucose, hypercholesterolemia, depression, stroke, cancer, asthma, chronic bronchitis or chronic obstructive pulmonary disease [COPD], and arthritis), as well as overweight status, smoking behavior (current or former), and alcohol consumption risk level (abstinent, low, medium, or high). Regional variation analyses were also performed within the canton of Vaud, comparing rural, semi-rural, and urban areas, with stratification by sex.

### Statistical Analysis

The prevalence of individuals meeting lung cancer screening eligibility criteria was estimated separately for each dataset, with corresponding 95% confidence intervals (CIs). Confidence intervals were calculated based on observed proportions and sample sizes within each cohort. Analyses of SHS data incorporated survey weights provided by the Federal Statistical Office to ensure representativeness of the canton of Vaud population.

For the Lc65+ cohort, smoking-related variables were sufficiently complete to compute eligibility without additional assumptions.

In the CoLaus dataset, smoking-related variables were collected differently for current and former smokers, resulting in structurally incomplete information on either smoking duration or daily consumption. To derive complete indicators of tobacco exposure, including pack-years,a targeted single-imputation strategy was applied. For participants who were former smokers, information on smoking duration was available whereas daily consumption was not systematically collected; for participants who were currently smoking, the inverse applied. Missing value for the age at which participants who were currently smoking had started was estimated based on the distribution observed among participants who were former smokers. This distribution was skewed, with values concentrated around the median, and formal normality tests (e.g., sktest) confirmed a significant deviation from normality. To minimize the influence of extreme values and ensure a conservative, robust approach, the median age of initiation (18 years) observed among former smokers was used as a single imputed value for all current smokers with missing data. This strategy aimed to reduce potential bias while maintaining comparability between smoking-status groups. The variable representing the average number of cigarette packs smoked per day had a high proportion of missing data among participants who were former smokers. To preserve the coherence of tobacco exposure indicators and maintain empirical variability, a stochastic imputation approach was applied. Specifically, bootstrap sampling with replacement was performed using the observed distribution of the average number of cigarette packs smoked per day among participants who were currently smoking, whose data were complete and considered representative of all smokers. Each former smoker with missing data was assigned a randomly selected value drawn from this empirical distribution, truncated at three packs per day, the maximum observed among current smokers. All imputations were implemented without modifying of observed values, allowing consistent calculation of smoking exposure indicators, including cumulative duration, daily consumption, and pack-years.

Some assumptions were made for missing values in the SHS. When smokers did not report their daily consumption of a specific tobacco product (cigarette, pipe, cigarillo, cigar, etc.) this value was assumed to be zero. Missing data for other eligibility-related variables were rare (0.06% for smoking status; 2.6% for number years of smoking; 0.06% for time since quitting) and simply treated as non-eligible. For variables used in bivariate comparisons, missing values were grouped under an “unknown” category.

In each of the three datasets, participants were classified as eligible or non-eligible for lung cancer screening based on the 2021 USPSTF criteria. Within each cohort, eligible and non-eligible participants were compared for clinical and sociodemographic characteristics. Because data collection methods varied across sources, comparisons were performed separately for each dataset, as specified in the corresponding cohort descriptions.

Continuous variables were summarized as means with standard deviations or medians with interquartile ranges, as appropriate. Categorical variables were described using frequencies and percentages. For univariate analyses, associations between categorical variables were assessed using the chi-square test or Fisher’s exact test, depending on cell counts. Differences in means were evaluated using the unpaired t-test for normally distributed data, and differences in medians were assessed using the Mann–Whitney U test for non-normally distributed data.

Univariate logistic regression was used to explore associations between screening eligibility and selected covariates.

A *p*-value <0.05 was considered statistically significant. All analyses were conducted using SAS version 9.4 (SAS Institute, Cary, NC) and R version 4.2.0 (R Foundation for Statistical Computing, Vienna, Austria).

## Results

The Lc65+ cohort included 1,678 participants aged 65–70 years, with 56.2% women and 43.8% men (Table 1). About one-quarter (25.2%) had a smoking history of more than 20 pack-years, and 18.2% (95% CI: 16.6%–20.3%) met the eligibility criteria for lung cancer screening. Among those with more than 20 pack-years of smoking, just over one-quarter (27.9%) had quit more than 15 years earlier and were therefore ineligible. Among the eligible individuals, more than half (58.7%) were still smoking.

**TABLE 1:** Comparison of characteristics by lung cancer screening eligibility according to 2021 U.S. Preventive Services Task Force criteria in the Lausanne cohort 65+ Study (Lausanne, Switzerland, 2014).

Category	All participants n = 1,678 (%)	Non-eligible n = 1,373 (%)	Eligible n = 305 (%)	p-value
Sex				
Female	943 (56.2)	783 (57.0)	160 (52.5)	0.15
Male	735 (43.8)	590 (43.0)	145 (47.5)	​
Age (years), mean (SD)	67.9 (1.41)	68.0 (1.4)	67.9 (1.4)	0.80
Born in Switzerland	Not specified	1,159 (84.4)	239 (78.4)	0.03
Monthly income (CHF^a^) Median (SD)	5,543.24 (3,751.8)	5,796.3 (3,807.6)	4,383.0 (3,247.1)	<0.01
Financial assistance				
Financial difficulties	198 (11.8)	146 (10.6)	52 (17.0)	<0.01
Subsidy for insurance	282 (16.8)	205 (14.9)	77 (25.2)	<0.01
Additional welfare	230 (13.7)	167 (12.2)	63 (20.7)	<0.01
Smoking status	​	​	​	<0.01
Current	321 (19.1)	142 (10.3)	179 (58.7)	​
Former	699 (41.7)	573 (41.7)	126 (41.3)	​
Never	650 (38.7)	650 (47.3)	0 (0.0)	​
>20 pack-years	423 (25.2)	118 (8.6)	305 (100.0)	<0.01
Alcohol problems	98 (5.8)	59 (4.3)	39 (12.8)	<0.01
Comorbidities				
Overweight	377 (22.5)	354 (25.8)	93 (30.5)	0.24
Hypertension	678 (40.4)	547 (39.8)	131 (43.0)	0.60
Hypercholesterolemia	570 (34.0)	448 (32.6)	122 (40.0)	0.04
Coronary heart disease	105 (6.3)	75 (5.5)	30 (9.8)	0.02
Stroke	61 (3.6)	41 (3.0)	20 (6.6)	0.01
Diabetes	163 (9.7)	125 (9.1)	38 (12.5)	0.21
Previous cancer	211 (12.6)	169 (12.3)	42 (13.8)	0.75
Depression	248 (14.8)	190 (13.8)	58 (19.0)	0.06
Arthrosis	540 (32.2)	440 (32.0)	100 (32.8)	0.96
None of the above	213 (12.7)	184 (13.4)	29 (9.5)	0.16
Feeling tired (last 4 Weeks)	​	​	​	0.04
No at all	960 (57.2)	806 (58.7)	154 (50.5)	​
A little	602 (35.8)	475 (34.6)	127 (41.6)	​
A lot	91 (5.4)	70 (5.1)	21 (6.9)	​
Education	​	​	​	0.01
Basic compulsory school	271 (16.2)	216 (15.7)	55 (18.0)	​
Apprenticeship	638 (38.0)	508 (37.0)	130 (42.6)	​
High school diploma	134 (7.9)	103 (7.5)	31 (10.2)	​
Professional diploma	254 (15.1)	213 (15.5)	41 (13.4)	​
University/College degree	378 (22.5)	330 (24.0)	48 (15.7)	​

^a^ CHF, Swiss Franc: 1 CHF, 1.26 $US, 1.08€ (November 2025).

SD, standard deviation.

P-values were obtained using the unpaired t-test for normally distributed continuous variables and the Mann–Whitney U test for non-normally distributed continuous variables, and the Pearson chi-square test or Fisher’s exact test for categorical variables, as appropriate.

The CoLaus cohort included 3,839 participants aged 50–79 years, with 55.1% women and 44.9% men (Table 2). More than half (54.9%) reported current or past use of cigarettes. One in five (20.1%) had a smoking history of at least 20 pack-years, and 16.0% (95% CI: 13.3%–19.0%) met the eligibility criteria for lung cancer screening. Among those eligible, nearly two-thirds (64.9%) were still actively using tobacco.

**TABLE 2:** Comparison of self-reported comorbidities by lung cancer screening eligibility according to 2021 U.S. Preventive Services Task Force criteria in the CoLaus Study (Lausanne, Switzerland, 2014–2017).

Variable	Overall participants n = 3,839 (100%)	Non-Eligible n = 3,224 (84.0%)	Eligible n = 615 (16.0%)	p-value
Sex				
Female Male	2,115 (55.1) 1724 (44.9)	1805 (56.0) 1,419 (44.0)	310 (50.4) 305 (49.6)	0.01
Age (years) mean (SD)	63.30 (8.12)	63.39 (8.2)	62.79 (7.6)	0.09
Birth in Switzerland	2,406 (62.7)	2009 (62.3)	397 (64.6)	0.29
Smoking status: Current Former Never	647 (16.9) 1,377 (35.9) 1815 (47.3)	248 (7.7) 1,161 (36.0) 1815 (56.3)	399 (64.9) 216 (35.1) 0	<0.01
Alcohol consumption	2,737 (71.3)	2,264 (70.2)	473 (76.9)	0.36
Comorbidities				
BMI (kg/m2) - mean (SD)	26.49 (4.74)	26.49 (4.8)	26.49 (4.7)	0.99
Hypertension	1,237 (32.2)	1,026 (31.8)	211 (34.3)	0.30
Hypercholesterolemia	1,325 (34.5)	1,072 (33.3)	253 (41.1)	<0.01
Metabolic syndrome	957 (24.9)	768 (23.8)	189 (30.7)	<0.01
Diabetes	397 (10.3)	326 (10.1)	71 (11.5)	0.11
Sleep apneas	223 (5.8)	188 (5.8)	35 (5.7)	0.92
Health parameters				
Abdominal obesity (WHO)^a^	1,368 (35.6)	1,127 (35.0)	241 (39.2)	0.03
HDL cholesterol (mmol/L) - mean (SD)	1.60 (0.47)	1.62 (0.47)	1.50 (0.46)	<0.01
LDL cholesterol (mmol/L) - mean (SD)	3.19 (0.91)	3.20 (0.90)	3.12 (1.0)	0.04
Triglycerides (mmol/L) - mean (SD)	1.31 (0.97)	1.28 (0.9)	1.49 (1.5)	<0.01
Systolic blood pressure (mmHg)- mean (SD)	127.35 (17.87)	127.50 (17.9)	126.60 (17.8)	0.27
Diastolic blood pressure (mmHg) - mean (SD)	77.79 (10.72)	77.91 (10.7)	77.10 (10.6)	0.10
Self-reported health rating				
Health rating: Very good Good Bad Very bad	830 (21.6) 2,135 (55.6) 84 (2.2) 8 (0.2)	738 (22.9) 1809 (56.1) 72 (2.2) 6 (0.2)	92 (15.0) 326 (53.0) 12 (2.0) 2 (0.3)	<0.01
Feeling tired: Not at all A little A lot	2082 (54.2) 1,302 (33.9) 408 (10.6)	1787 (55.4) 1,069 (33.2) 325 (10.1)	295 (48.0) 233 (37.9) 83 (13.5)	<0.01
Difficulty of taking care of yourself: No difficulty at all I Had difficulty but I managed on my own I Needed help	3,533 (92.0) 199 (5.2) 59 (1.5)	2,973 (92.2) 154 (4.8) 53 (1.6)	560 (91.1) 45 (7.3) 6 (1.0)	0.02
Losing weight last 12 months	396 (10.3)	310 (9.6)	86 (14.0)	<0.01

SD, standard deviation.

^a^ According to the World Health Organization (WHO), abdominal (central) obesity is defined by waist circumference.

P-values were obtained using the unpaired t-test for normally distributed continuous variables and the Mann–Whitney U test for non-normally distributed continuous variables, and the Pearson chi-square test or Fisher’s exact test for categorical variables, as appropriate.

The SHS data were weighted to represent 253,976 adults aged 50–79 years residing in the canton of Vaud (Table 3). Just over half (52.4%) had reported current or past use of cigarettes. Among current smokers, 8.7% had a history of at least 20 pack-years, and among former smokers, 8.7% had quit within the past 10 years and also met the pack-year criterion. Overall 14.4% (95% CI: 11.7%–17.1%) met the eligibility criteria for lung cancer screening according to the 2021 USPSTF definition.

**TABLE 3:** Comparison of sociodemographic characteristics and self-reported comorbidities by lung cancer screening eligibility according to 2021 U.S. Preventive Services Task Force criteria in the Swiss Health Survey (canton of Vaud, Switzerland, 2022; weighted population = 253 976).

Variable	All participants N = 253,976	Non-eligible N = 217,384	Eligible N = 36,592	p-value
Age, mean (SD)	62 (8)	62 (9)	60 (7)	<0.01
Sex	​	​	​	<0.01
Female	135,597 (53.4%)	120,637 (55.5%)	14,960 (40.9%)	​
Male	118,379 (46.6%)	96,747 (44.5%)	21,632 (59.1%)	​
Nationality	​	​	​	0.70
Swiss	202,245 (79.6%)	172,624 (79.4%)	29,621 (80.9%)	​
Other	51,731 (20.4%)	44,760 (20.6%)	6,971 (19.1%)	​
Civil status	​	​	​	<0.01
Single	28,116 (11.1%)	24,762 (11.4%)	3,354 (9.2%)	​
Married	159,133 (62.7%)	142,032 (65.3%)	17,102 (46.7%)	​
Widowed, divorced, separated	66,727 (26.3%)	50,590 (23.3%)	16,137 (44.1%)	​
Monthly household income (CHF^a^)	​	​	​	0.30
<4,500	9,874 (14.3%)	7,637 (12.9%)	2,237 (23.5%)	​
4,500–5,999	15,561 (22.6%)	14,404 (24.3%)	1,157 (12.1%)	​
6,000–9,000	16,905 (24.5%)	14,631 (24.7%)	2,273 (23.9%)	​
>9,000	26,527 (38.5%)	22,674 (38.2%)	3,853 (40.5%)	​
Unknown	185,110	158,038	27,071	​
Household size (number of persons)	​	​	​	<0.01
1	69,470 (27.4%)	54,079 (24.9%)	15,391 (42.1%)	​
2	113,522 (44.7%)	101,018 (46.5%)	12,504 (34.2%)	​
3	37,203 (14.6%)	32,081 (14.8%)	5,122 (14.0%)	​
4 or more	33,781 (13.3%)	30,206 (13.9%)	3,575 (9.8%)	​
Education	​	​	​	0.01
Compulsory school	27,512 (11.1%)	23,454 (11.1%)	4,058 (11.3%)	​
Upper secondary level	114,422 (46.3%)	92,954 (44.0%)	21,467 (60.0%)	​
Tertiary level	105,229 (42.6%)	94,952 (44.9%)	10,277 (28.7%)	​
Unknown	6,813	6,024	789	​
Professional situation	​	​	​	0.20
Not working, retired	116,113 (45.7%)	101,905 (46.9%)	14,208 (38.8%)	​
Unemployed	5,158 (2.0%)	3,756 (1.7%)	1,402 (3.8%)	​
Part-time work	49,634 (19.5%)	43,310 (19.9%)	6,323 (17.0%)	​
Full-time work (90%–100%)	83,071 (32.9%)	68,413 (31.5%)	14,659 (40.1%)	​
Health parameters and self-reported comorbidities				
Self-rated health	​	​	​	0.20
Excellent	80,363 (31.8%)	71,084 (32.9%)	9,279 (25.4%)	​
Good	121,389 (48.0%)	103,238 (47.8%)	18,151 (49.6%)	​
Average, poor	50,927 (20.2%)	41,766 (19.3%)	9,161 (25.0%)	​
Long-term illness/health problem	149,187 (58.7%)	125,157 (58.0%)	24,030 (66.3%)	0.12
Hypertension	12,772 (5.2%)	11,712 (5.4%)	1,060 (3.0%)	0.30
High glucose level	8,989 (3.9%)	7,269 (3.6%)	1,720 (5.4%)	0.40
High cholesterol level	23,343 (10.0)	17,838 (9.1%)	5,506 (17.1%)	0.03
Depression (last 12 months)	18,964 (7.5%)	12,712 (5.9%)	6,252 (17.3%)	<0.01
Stroke (lifetime)	6,705 (2.6%)	4,692 (2.2%)	2,013 (5.5%)	<0.05
Cancer (lifetime)	27,179 (10.7%)	22,703 (10.5%)	4,477 (12.6%)	0.6
Asthma (last 12 months)	14,023 (5.5%)	10,227 (4.7%)	3,796 (10.5%)	0.03
Chronic bronchitis, COPD, emphysema (last 12 months)	8,477 (3.3%)	6,039 (2.8%)	2,439 (6.8%)	0.06
Arthritis (last 12 months)	17,382 (6.9%)	14,353 (6.7%)	3,029 (8.4%)	0.50
Overweight	123,179 (48.5%)	106,824 (49.3%)	16,356 (45.5%)	0.50
Smoking status (past vs. current)	​	​	​	<0.01
Former	81,361 (61.1%)	66,778 (69.2%)	14,583 (39.9%)	​
Current	51,765 (38.9%)	29,756 (30.8%)	22,009 (60.1%)	​
Unknown	120,850	120,850	0	​
Alcohol consumption - risk level	​	​	​	<0.01
Abstinent	39,189 (15.4%)	34,714 (16.0%)	4,475 (12.2%)	​
Low risk	167,508 (66.0%)	147,342 (67.8%)	20,166 (55.1%)	​
Medium or high risk	47,279 (18.6%)	35,328 (16.3%)	11,951 (32.7%)	​

^a^ CHF, Swiss Franc: 1 CHF, 1.22 $US, 1.07€ (June 2025).

P-values were obtained using the unpaired t-test for normally distributed continuous variables and the Mann–Whitney U test for non-normally distributed continuous variables, and the Pearson chi-square test or Fisher’s exact test for categorical variables, as appropriate. Survey weights were applied as described in the Methods.

### Lung Cancer Screening Eligibility by Age and Sex

**FIGURE 1:**
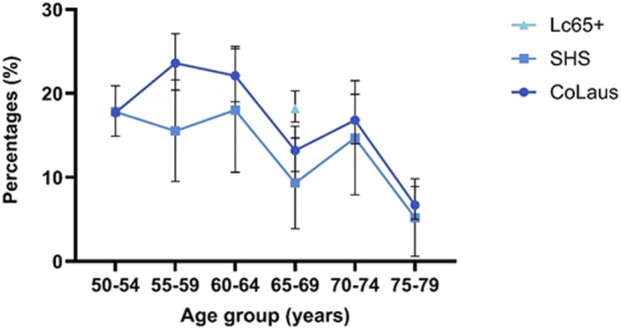
Eligibility for lung cancer screening in the canton of Vaud, Switzerland, by age group, according to the 2021 United States Preventive Services Task Force criteria (95% confidence intervals). Comparative estimates are shown from three data sources: Lausanne Cohort 65+ (2014, ages 65–70); CoLaus study (2014–2017, ages 50–79, smoking history modeled); and Swiss Health Survey (2022, ages 50–79). The figure illustrates the proportion of eligible individuals across datasets and age groups.

Eligibility for lung cancer screening varied by age across the three datasets (Figure 1; Supplementary Table S1). In Lc65+, which only included individuals aged 65 to 69, the overall eligible fraction was slightly higher than for the other data sources.

In CoLaus, eligibility fraction was highest among participants in their late 50s for both sexes, then declined progressively with age, reaching its lowest value in those aged 75 to 79. A similar pattern was seen in the SHS, although eligibility peaked slightly later, in the 60–64 age group, before decreasing in older age groups.

**FIGURE 2:**
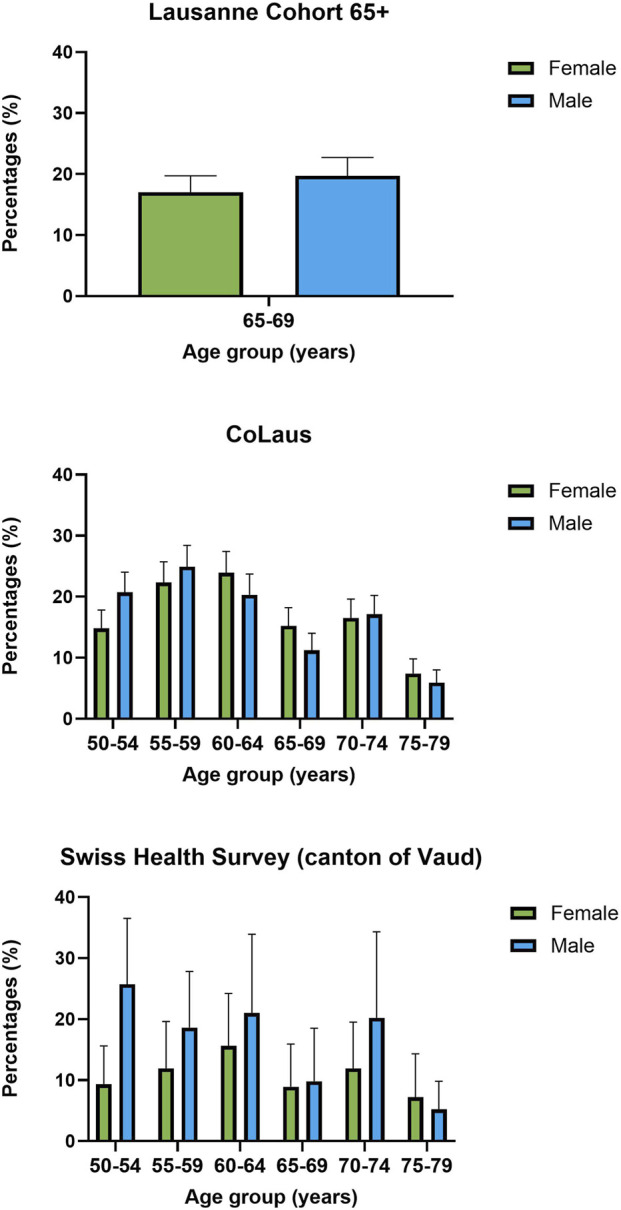
Eligibility for lung cancer screening in the canton of Vaud, Switzerland, by age group and sex, according to the 2021 United States Preventive Services Task Force criteria (95% confidence intervals). Panel A shows the Lausanne Cohort 65+ (2014, residents aged 65–70); Panel B shows the CoLaus study (2014–2017, Lausanne residents aged 50–79, with smoking history modeled for missing data); and Panel C shows the Swiss Health Survey (2022, Vaud residents aged 50–79, using a 10-year instead of 15-year quitting period).

Across all age groups and datasets, men were consistently more likely to meet the screening criteria than women (Figure 2). In Lc65+, eligibility was slightly, but not statistically significantly higher in men (19.7% vs. 17%). In both CoLaus and SHS, however, the sex difference was more pronounced, with men showing significantly higher rates of eligibility (CoLaus: 17.7% vs. 12.2%, *p* = 0.011; SHS: 18.3% vs. 11.0%, *p* = 0.007).

The disparity was most pronounced in the youngest age group (50–54 years) in the SHS, where eligibility among men was nearly three times higher than among women (25.7% vs. 9.3%). Similar, though smaller, gaps were observed in the 55–59-year age group, with higher eligibility among men than women in both CoLaus (24.9% vs. 22.3%) and SHS (18.6% vs. 11.9%). This sex pattern reversed slightly in the oldest participants (75–79 years), with eligibility rates being marginally higher among women than men in both CoLaus (7.4% vs. 5.9%) and SHS (7.2% vs. 5.2%).

### Characteristics of the Population Eligible for Lung Cancer Screening by Data Source

#### Lausanne Cohort 65+ (Lc65+)

In the Lc65+ dataset, 65–69-year-old individuals eligible for lung cancer screening had significantly higher prevalences of comorbidities compared to non-eligible individuals, including hypercholesterolemia, coronary heart disease, and alcohol-related problems (*all p* < 0.05; Table 1). They were also more likely to report feeling somewhat or very fatigued (*p* < 0.05).

**FIGURE 3:**
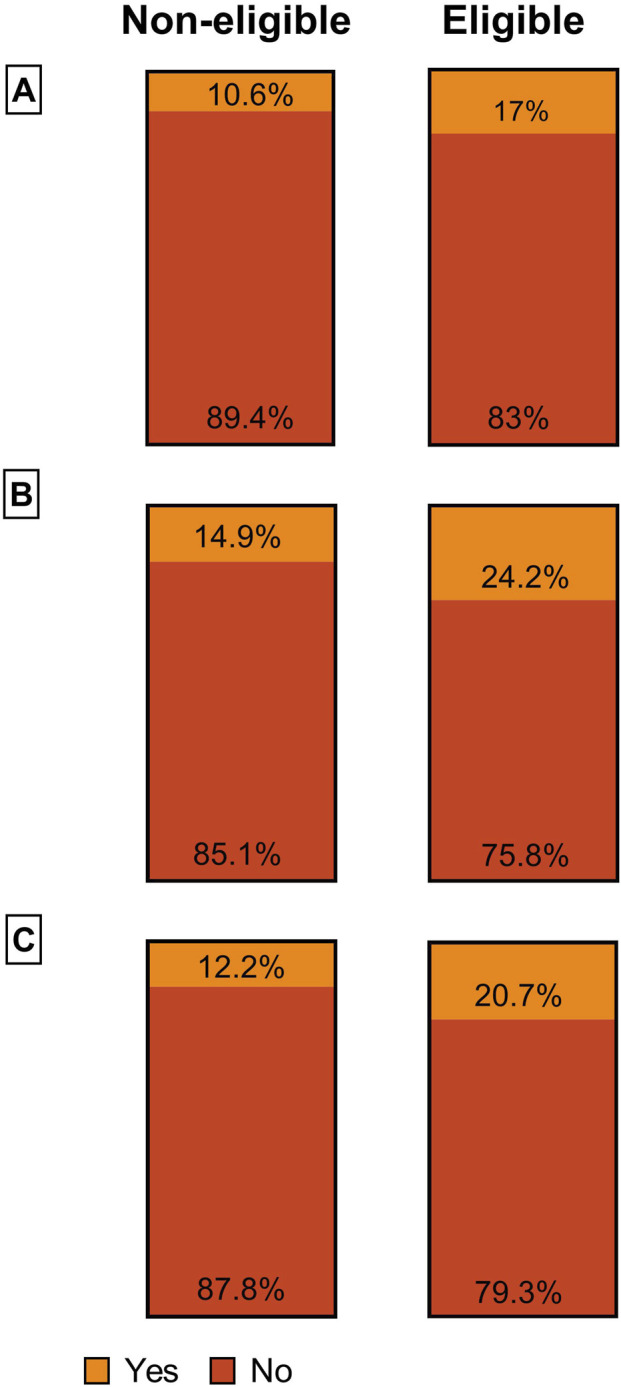
Financial characteristics of screening-eligible and non-eligible participants in the Lausanne Cohort 65+ Study (Lausanne, Switzerland, 2014). Distribution of self-reported financial indicators among individuals eligible and not eligible for lung cancer screening according to the 2021 U.S. Preventive Services Task Force (USPSTF) criteria. The figure displays six pie charts grouped into three panels: **(A)** Financial difficulties, **(B)** Subsidy for health insurance, and **(C)** Additional welfare benefits. All differences were statistically significant (p < 0.01).

Significant socioeconomic disparities were observed. Eligible individuals reported significantly lower monthly incomes and were more likely to receive health insurance subsidies or additional welfare support (Figure 3; *all p* < 0.01). They were also less likely to have been born in Switzerland and had lower levels of educational attainment.

#### CoLaus|PsyCoLaus

In the CoLaus dataset, significant health differences were observed between individuals eligible and non-eligible for lung cancer screening (Table 2). Eligible individuals reported poorer self-rated health and a higher prevalence of metabolic conditions, including metabolic syndrome and hypercholesterolemia (both *p* < 0.01). Other comorbidities—such as hypertension, diabetes, depression, prior cancer, and arthrosis—did not differ significantly between groups. Eligible participants also had a significantly higher prevalence of abdominal obesity, lower HDL cholesterol, higher triglyceride levels, and slightly elevated LDL cholesterol concentrations (*all p* < 0.05).

Regarding self-reported health indicators, eligible individuals were more likely to report fatigue, difficulty with self-care, and recent weight loss (*all p* < 0.05). They were also less likely to rate their health as “very good” (*p* < 0.001).

#### Swiss Health Survey

The weighted analysis of the 2022 SHS data revealed significant differences in health status and sociodemographic characteristics between individuals eligible and non-eligible for lung cancer screening (Table 3). From a health perspective, eligible individuals reported a significantly higher prevalence of several comorbidities, including high cholesterol, depression, stroke, asthma. And were also more likely to engage in medium to high-risk alcohol consumption behaviors (*all p* < 0.05).

In terms of sociodemographic characteristics, eligible participants were slightly younger (mean age: 60 vs. 62 years), more often male, and more frequently lived alone or reported being widowed, divorced, or separated (*all p* < 0.05). They were less likely to have completed tertiary education and tended to live in smaller households (*p* < 0.05). No significant differences were observed in income levels or employment status.

The SHS data were not limited to Lausanne but represented the entire canton of Vaud. Eligibility varied by region type and sex (Supplementary Table S2). Among men, the highest eligibility was observed in rural areas, followed by urban, and semi-rural areas. Among women, eligibility was highest in semi-rural areas, followed by urban and rural settings. However, these differences were not statistically significant.

## Discussion

This study assessed lung cancer screening eligibility in Lausanne and Vaud residents, Switzerland, using 2021 USPSTF criteria and three independent population-based datasets: Lc65+, CoLaus, and the Vaud subset of the 2022 SHS. Eligibility rates overall ranged from 14.4% in the SHS to 16.0% in CoLaus and 18.2% in Lc65+, offering consistent estimates despite differences in sampling, data collection periods and areas covered. The lowest eligibility fraction observed in the SHS may reflect recent declining tobacco use, lower rural smoking prevalence, and the use of a 10-year cessation cut-off in contrast to the 15-year threshold used in the urban-based Lc65+ and CoLaus cohorts. The decline over time in the proportion of people who smoke >20 cigarettes a day in Switzerland is consistent with our results based on different times of data collection in our sources — Lc65+ in 2014, CoLaus in 2014–2017, and SHS in 2022 [23]. Our findings align with U.S. and French estimates under the 2021 USPSTF guidelines, which suggest that 14%–23% and 17.3% of American and French adults in the target age group are eligible, respectively [24–26].

The high concordance observed across the three data sources substantiates the generalizability of our findings to the population of the canton of Vaud and, more broadly, to Switzerland. Translating these proportions—approximately one in six individuals—into absolute numbers, approximately 44,627 residents among 267,672 of the Vaud population, aged 50–79 years in 2024, would be potentially eligible for lung cancer screening [27]. At this scale, screening would entail substantial costs, including repeated LDCT scans, clinical assessments, smoking cessation services, and downstream procedures, yet modeling studies suggest it can be cost-effective in high-income countries with a significant smoking burden such as Switzerland [28, 29].

Eligibility peaked around ages 55–64 before declining in older individuals, potentially due to smoking cessation beyond 15-year or higher mortality among long-term smokers. Across all cohorts, men were more frequently eligible than women—except in the oldest age group—a pattern consistent with sex differences in tobacco use and survival in this region [30, 31]. A large U.S. study confirmed that in Western countries women had lower odds of meeting the 2021 USPSTF criteria, with similar patterns observed in modeling work from France [26].

Screening eligibility criteria are central to determining which individuals are identified as being at sufficiently high risk to benefit from lung cancer screening. We used the 2021 USPSTF criteria, which expanded the 2013 version by lowering the age to 50 and the smoking history to 20 pack-years [32]. While 2021 USPSTF criteria are simpler to apply, they may overlook important risk factors [33]. In contrast, risk-based models like PLCOm2012 place greater emphasis on increasing age and incorporate multiple factors, such as COPD, family history, race/ethnicity, pollution exposure, and socioeconomic status, resulting in greater sensitivity [33]. However, the application of such models requires detailed individual-level data that were not consistently available across the datasets used in this study. As a result, we focused on USPSTF criteria, which are widely implemented, operationally straightforward, and directly aligned with the current screening pilot in the canton of Vaud.

Although included in the 2021 USPSTF criteria, the smoking cessation criterion remains debated. The American Cancer Society recently recommended against excluding former smokers based solely on time since quitting [34]. Removing this 15-year threshold would expand eligibility from 18.2% to 25.2%in Lc65+, and from 16.0% to 20.1% in CoLaus, significantly increasing access.

A consistent finding across all three cohorts was the high proportion of current smokers among those eligible for screening: 58.0% (Lc65+), 64.9% (CoLaus), and 60.1% (SHS). These figures highlight the critical importance of integrating smoking cessation consultation into screening programs [35, 36]. Notably, the NLST reported a 38% reduction in lung cancer mortality when screening was paired with smoking cessation [37].

Eligible individuals in all three cohorts had a greater burden of comorbidities, including dyslipidemia, coronary heart disease, metabolic syndrome, depression, and high-risk alcohol consumption. These findings were reinforced by clinical indicators such as abdominal obesity, adverse lipid profiles, poorer self-rated health, fatigue, and unintentional weight loss, reflecting a substantial overall health burden. A recent meta-analysis confirmed that comorbidities are highly prevalent among lung cancer screening-eligible populations, often exceeding levels seen in other cancer screening programs [38]. These risk factors are reflected in the high prevalence of coronary calcifications and emphysema on LDCT in lung cancer screening programs, which ultimately correlate with long-term mortality [39–41]. These data underscore the need to embed lung cancer screening within comprehensive, person-centered care frameworks. Screening should serve not only as a tool for early cancer detection but also as an entry point for broader health interventions, including health promotion, behavioral support, and post-treatment surveillance—ultimately addressing the complex needs of this vulnerable population [38, 42–46]. Nevertheless, the best means of integrating this information into routine prevention provided in primary care is not yet known.

Notably, the less healthy participants in our study likely represent a healthier subset than the general population, partly due to non-response bias, potentially leading to an underestimation of the true burden among screening-eligible individuals [47].

Socio-demographic differences between eligible and non-eligible individuals are marked: in the Lc65+ cohort, 25.2% of eligible individuals received health insurance subsidies, compared to 14.9% of non-eligible individuals. Eligibility was also associated with lower income and education. Social disparities were also evident in the SHS data, where eligible individuals were more likely to be widowed, divorced, or living alone, and had lower educational attainment. These disparities highlight the need for equitable, accessible lung cancer screening strategies. Reaching this vulnerable population will require simplified access and integration with targeted public health efforts to reduce social inequalities [5]. Comparable disparities have been documented in other settings, including in England, where a population-based study linked lung cancer incidence and diagnosis to ethnicity, socioeconomic status, and other demographic factors [48].

The financial vulnerability of screening-eligible individuals deserves close attention, particularly given the implementation challenges of lung cancer screening. Current participation remains low with 16% of eligible individuals in the U.S. being screened [49, 50]. A meta-analysis of 21 studies found adherence to follow-up screening ranged from 46% to 69%, far below the 95% reported in the NLST [49]. Strategies to increase uptake include full financial coverage, improved accessibility, and strengthened communication between healthcare professionals and community leaders [5, 51].

We provided the first population-based estimates of lung cancer screening eligibility in a Swiss region—offering novel data that are critical for planning the implementation of screening programs in Switzerland [52]. By comparing across three rich data sets sampling from the Lausanne (Vaud) population, we were able to provide more robust estimates and multiple comparisons with the non-eligible population. Further, all three cohorts specifically invited a representative cross-section of the local population. These results provided relevant background for the ongoing Vaud pilot program on lung cancer screening and may help inform future implementation strategies. Future analyses on the Vaud lung cancer screening program will provide further insights on eligibility in Switzerland.

### Limitations

Several limitations must be acknowledged. Two datasets were collected several years ago (Lc65+ in 2014 and CoLaus in 2014–2017), and all relied on self-reported smoking data, which is subject to recall bias. In CoLaus, pack-years for current smokers were imputed using the median age at smoking initiation from former smokers, assuming similar distributions between groups; this simplification may reduce precision in eligibility estimates. In the SHS, the cessation window was limited to 10 years, which underestimates eligibility according to USPSTF criteria. Some community-dwelling adults—although technically eligible based on age and smoking history—may not be candidates for curative surgery because of significant comorbidities or impaired pulmonary function. This limitation is difficult to address without objective lung function assessments. Such individuals are often excluded from clinical trials, leaving the benefit of screening in this subgroup uncertain, despite encouraging outcomes reported with ablative radiotherapy [53, 54]. However, this limitation is likely mitigated by the fact that survey participants tend to be somewhat healthier than the general population, especially in the CoLaus cohort that had significant loss to follow-up. This may lead to underestimation of the number of individuals eligible for screening.

Due to the aforementioned data limitations, we could not verify eligibility rates based on individual risk prediction models (such as PLCOm2012 or LLP), neither compare them to the USPSTF eligibility criteria used here. Incorporating such models in future analyses could help better assess their applicability and potential utility in the Swiss context. Crude comparisons of eligibility rates between datasets are also potentially biased due to differing age ranges and inclusion criteria across cohorts, which should be considered when interpreting the results.

Future research should assess whether lung cancer patients in Vaud would have met current screening criteria and explore cost-effectiveness to guide locally adapted protocols. At the same time, the focus may also need to shift toward the thoughtful implementation of a lung cancer screening program—while continuing to monitor eligibility patterns and evaluate whether existing criteria should be adapted. If Switzerland were to move toward implementing lung cancer screening, particular attention should be given to promoting equitable access, especially for higher-risk populations who may face structural barriers to care.

### Conclusion

Our study tackles the challenge of lung cancer screening eligibility in a Swiss population—an essential step toward implementing organized screening. Based on the 2021 USPSTF criteria, approximately one in six individuals aged 50 to 79 (14.4%–18.2%) would qualify for screening, most of whom are males and currently smoking. Across three independent data sources, our findings consistently show that eligible individuals face greater health risks and socioeconomic disadvantages. Taken together, these results reveal that a substantial portion of the population could benefit from lung cancer screening, and that their heightened medical and social vulnerability calls for a comprehensive, person-centered approach—combining screening with tobacco cessation support, health promotion, and equitable access to care.

## Data Availability

Lc65+ data are available upon reasonable request. The data that support the findings of this study are deidentified participant data. Details of the data and how to request access are available from the principal investigator of the Lc65+ study (yves.henchoz@unisante.ch) at the Centre for Primary Care and Public Health (Unisanté), University of Lausanne, Switzerland. The data of CoLaus|PsyCoLaus study used in this article cannot be fully shared as they contain potentially sensitive personal information on participants. According to the Ethics Committee for Research of the Canton of Vaud, sharing these data would be a violation of the Swiss legislation with respect to privacy protection. However, coded individual-level data that do not allow researchers to identify participants are available upon request to researchers who meet the criteria for data sharing of the CoLaus|PsyCoLaus Datacenter (CHUV, Lausanne, Switzerland). Any researcher affiliated to a public or private research institution who complies with the CoLaus|PsyCoLaus standards can submit a research application to research.colaus@chuv.ch or research.psycolaus@chuv.ch. Proposals requiring baseline data only, will be evaluated by the baseline (local) Scientific Committee (SC) of the CoLaus and PsyCoLaus studies. Proposals requiring follow-up data will be evaluated by the follow-up (multicentric) SC of the CoLaus|PsyCoLaus cohort study. Detailed instructions for gaining access to the CoLaus|PsyCoLaus data used in this study are available at www.colauspsycolaus.ch/professionals/how-to-collaborate/. The study protocol and the Stata code used to analyze the data can be provided by the corresponding author upon reasonable request. The data from the 2022 Swiss Health Survey were provided by the Federal Statistical Office (FSO). In accordance with applicable regulations, the recipient (JB) may use the data solely on their own behalf and may engage auxiliary personnel under their direct supervision and responsibility, ensuring full compliance with all legal and contractual obligations. Granting third-party access or transmitting the data in any form is strictly prohibited unless explicitly authorized by the FSO.
