# Supplementary material for: Eligibility for Lung Cancer Screening in Switzerland: A Comparative Analysis of Three Data Sources From Lausanne and the Canton of Vaud
Source: Int J Public Health. 2026 Jan 21;71:1609104. doi: 10.3389/ijph.2026.1609104 (PMC12867931; doi:10.3389/ijph.2026.1609104)
Supplement: Supplementary file 1 [file Supplementaryfile1.docx]

**Supplementary Material**

**Table S1. Eligibility for Lung Cancer Screening by Age and Sex in the Lausanne Cohort 65+ (Lc65+), CoLaus|PsyCoLaus, and the Vaud Subsample of the Swiss Health Survey (SHS)**

| Age Group (years) | CoLaus (%) | CI 95% | SHS  (%) | CI 95% | Lc65+  (%) | CI 95% |
| --- | --- | --- | --- | --- | --- | --- |
| 50-54 |  |  |  |  |  |  |
| Total | **17.7** | [14.9; 20.9] | **17.8** | [14.9; 20.9] |  |  |
| Female | **14.8** | [12.2; 17.8] | **9.3** | [3; 15.6] |  |  |
| Male | **20.7** | [17.6; 24.0] | **25.7** | [14.9; 36.5] |  |  |
| 55-59 |  |  |  |  |  |  |
| Total | **23.6** | [20.4; 27.1] | **15.5** | [9.5; 21.6] |  |  |
| Female | **22.3** | [19.2; 25.7] | **11.9** | [4.2; 19.6] |  |  |
| Male | **24.9** | [21.6; 28.4] | **18.6** | [9.5; 27.8] |  |  |
| 60-64 |  |  |  |  |  |  |
| Total | **22.1** | [19.0; 25.6] | **18** | [10.6; 25.3] |  |  |
| Female | **23.9** | [20.7; 27.4] | **15.6** | [7; 24.2] |  |  |
| Male | **20.3** | [17.3; 23.7] | **21** | [8.2; 33.9] |  |  |
| 65-69 |  |  |  |  |  |  |
| Total | **13.2** | [10.7; 16.1] | **9.3** | [3.9; 14.7] | **18.2** | [16.6; 20.3] |
| Female | **15.2** | [12.5; 18.2] | **8.9** | [1.8; 15.9] | **17** | [14.9; 19.7] |
| Male | **11.2** | [9.0; 14.0] | **9.8** | [1.1; 18.5] | **19.7** | [17; 22.7] |
| 70-74 |  |  |  |  |  |  |
| Total | **16.8** | [14.0; 19.9] | **14.7** | [7.9; 21.5] |  |  |
| Female | **16.5** | [13.7; 19.6] | **11.9** | [4.4; 19.5] |  |  |
| Male | **17.1** | [14.3; 20.2] | **20.2** | [6; 34.3] |  |  |
| 75-79 |  |  |  |  |  |  |
| Total | **6.7** | [5.0; 8.9] | **5.2** | [0.6; 9.8] |  |  |
| Female | **7.4** | [5.7; 9.8] | **7.2** | [0.1; 14.3] |  |  |
| Male | **5.9** | [4.3; 8.0] | **5.2** | [0.6; 9.8] |  |  |
| Overall |  |  |  |  |  |  |
| Total | **16.0** | [13.3; 19.0] | **14.4** | [11.7; 17.1] | **18.2** | [16.6; 20.3] |
| Female | **14.7** | [12.1; 17.6] | **11** | [8; 14.3] | **17** | [14.9; 19.7] |
| Male | **17.7** | [14.9; 20.9] | **18.3** | [13.7; 22.9] | **19.7** | [17; 22.7] |

**Table S2. Lung Cancer Screening Eligibility by Rurality, Vaud Subsample of the Swiss Health Survey (SHS)**

| Type of region | Overall eligibility (%) (95% CI) | Eligibility (%) in Males (95% CI) | Eligibility (%) in Females (95% CI) |
| --- | --- | --- | --- |
| **Rural** | 15.47% (9.14-21.80) | 25.86% (13.80-37.93) | 7.28% (1.92-12.64) |
| **Semi-rural** | 16.19% (10.29-22.09) | 14.12% (5.73-22.51) | 18.16% (9.71-26.62) |
| **Urban** | 13.38% (9.92-16.83) | 17.49% (11.51-23.47) | 9.78% (6.03-13.54) |
| **Overall** | 14.41% (11.72-17.10) | 18.27% (13.71-22.84) | 11.03% (8.00-14.06) |
